# Supplementary material for: Capacity building efforts and perceptions for wildlife surveillance to detect zoonotic pathogens: comparing stakeholder perspectives
Source: BMC Public Health. 2014 Jul 4;14:684. doi: 10.1186/1471-2458-14-684 (PMC4096412; doi:10.1186/1471-2458-14-684)
Supplement: Additional file 1 — Rapid survey tool. [file 1471-2458-14-684-S1.pdf]

<u>Section 1. Wildlife Official</u>

I'm going to ask you about ways in which contact between wildlife and humans may be important for zoonotic pathogen transmission or risk of exposure to humans. For each of the following categories can you please tell me whether you think the type of contact is important for disease transmission (or exposure)?

- Hunting
- Butchering wildlife
- Eating wildlife
- Markets
- Crop raiding
- Wildlife living near human dwellings
- Wildlife-livestock interactions. Please describe.
- Animals in captivity (e.g. temporary holding facilities, private collections, farms, rehabilitation centers).
- Ecotourism
- Shared water sources
- Extraction areas (e.g. mining, oil/gas).
- Areas of ongoing land use change (e.g. deforestation, road building).
- Are there other important interfaces we should consider? Please list.

I have a list of global topics of challenges for conducting wildlife surveillance for zoonotic pathogens. Which of these do you think are important in the country?

- Lack of sustainable funding/resources
- Large country size/remoteness/limited infrastructure
- Insufficient human capacity
- Insufficient laboratory capacity
- Lack of existing government wildlife surveillance program or wildlife policies
- Insufficient communication or coordination among agencies, sectors, or regions
- Limited interest or awareness regarding wildlife or associated disease issues
- Cultural acceptability
- Detrimental consequences for tourism and exports
- Political issues
- Other:

I have a list of global topics of opportunities for improving wildlife surveillance for zoonotic pathogens. Which of these do you think are important in the country?

- Increasing funding
- Increasing human capacity from new/existing training programs
- Increasing laboratory capacity from new/existing programs and facilities

• Building on existing surveillance networks/programs<br>• Collaboration with local/foreign programs or organizations<br>• Growing interest or awareness regarding wildlife diseases or surveillance programs<br>• Increasing communication or coordination among agencies, sectors, or regions<br>• Wide availability of communication devices (e.g. cell phones, radios) and communication networks<br>• Using conservation to provide incentive for performing surveillance<br>• Political stability in the country<br>• Other:
**Have there been any known disease outbreaks in humans or livestock that may have come from wildlife in recent years? Was your organization involved? Please describe.**

<u>Section 2. Project Scientist</u>

**List the high priority interfaces that are important for zoonotic pathogen surveillance in wildlife in your country**<br><br>**Of the interfaces listed above, which interfaces are you working at in your country?**
**What do you see as the biggest challenges and opportunities for building capacity in your country to implement and sustain effective surveillance of zoonotic pathogens in wildlife populations?**
**Have there been any known disease outbreaks in livestock or people that were believed to have spilled over from wildlife since the PREDICT project began? Please describe:**
